# Public preference on sharing health data to inform research, health policy and clinical practice in Australia: A stated preference experiment

**DOI:** 10.1371/journal.pone.0290528

**Published:** 2023-11-16

**Authors:** Richard J. Varhol, Richard Norman, Sean Randall, Crystal Man Ying Lee, Luke Trevenen, James H. Boyd, Suzanne Robinson

**Affiliations:** 1 School of Population Health, Curtin University, Perth, Western Australia, Australia; 2 Deakin Health Economics, Institute for Health Transformation, Deakin University, Melbourne, Victoria, Australia; 3 School of Psychology and Public Health, La Trobe University, Melbourne, Australia; The University of Sydney, AUSTRALIA

## Abstract

**Objective:**

To investigate public willingness to share sensitive health information for research, health policy and clinical practice.

**Methods:**

A total of 1,003 Australian respondents answered an online, attribute-driven, survey in which participants were asked to accept or reject hypothetical choice sets based on a willingness to share their health data for research and frontline-medical support as part of an integrated health system. The survey consisted of 5 attributes: Stakeholder access for analysis (Analysing group); Type of information collected; Purpose of data collection; Information governance; and Anticipated benefit; the results of which were analysed using logistic regression.

**Results:**

When asked about their preference for sharing their health data, respondents had no preference between data collection for the purposes of clinical practice, health policy or research, with a slight preference for having government organisations manage, govern and curate the integrated datasets from which the analysis was being conducted. The least preferred option was for personal health records to be integrated with insurance records or for their data collected by privately owned corporate organisations. Individuals preferred their data to be analysed by a public healthcare provider or government staff and expressed a dislike for any private company involvement.

**Conclusions:**

The findings from this study suggest that Australian consumers prefer to share their health data when there is government oversight, and have concerns about sharing their anonymised health data for clinical practice, health policy or research purposes unless clarity is provided pertaining to its intended purpose, limitations of use and restrictions to access. Similar findings have been observed in the limited set of existing international studies utilising a stated preference approach. Evident from this study, and supported by national and international research, is that the establishment and preservation of a social license for data linkage in health research will require routine public engagement as a result of continuously evolving technological advancements and fluctuating risk tolerance. Without more work to understand and address stakeholder concerns, consumers risk being reluctant to participate in data-sharing and linkage programmes.

## Introduction

Medical information, including family histories, prescriptions and pathology reports are routinely collected and entered into primary-care-based Electronic Health Records (EHRs). Patients with specific diagnoses or requiring specialised procedures are subsequently referred to hospital-based specialists, where additional clinical and administrative data is gathered pertaining to service utilisation, procedures provided and patient outcomes, with most data stored in hospital EHRs, health insurance databases and registries (including cancer and notifiable diseases). However, management of patient’s medical data and enabling the provision of consent is a shortcoming of most EHRs often restricting access and use of their medical records for secondary use.

In Australia, as in many other parts of the world, issues associated with public trust, informed consent, and consideration of minority inclusion. Previous studies have shown a positive correlation between the level of public trust and the willingness to share health data for research purposes, however the degree varies across demographics and locality, necessitating context-specific studies to better understand regional dynamics [1–5].

In Australia, much of the healthcare system is siloed as a result of complex funding arrangements between state (acute care) and federal (primary care, radiology, pathology and medication) government [6]. Issues with the collection and integration of data are further exacerbated in the primary care setting where community-based medical practitioners use one of eight independently developed EHRs [7].

To enable meaningful insights across disparate EHRs and data collections, data linkage methodologies have been developed to bring together all records that belong to the same individual, family, place or event across multiple data sources [8]. In Australia, most state governments, and the Commonwealth, have established dedicated third-party ‘linkage units’, which are predominantly responsible for joining together health collections for research and policymaking. These services allow researchers to map an individual’s lifetime journey through the health system providing a valuable understanding of relationships between social factors and health status [9, 10], access to health services [11] and improving continuity of care [12–14].

Although data linkage is a widely used technique, it typically occurs without patient consent through the use of personal identifying information (PII) from health records which are made available to trusted third parties for data integration. Due to these data sensitivities, a range of privacy safeguards and governance processes exist around the transfer and use of health data. These include legal and contractual arrangements, policies governing data access [15–17], information security requirements [18, 19], and a specific privacy-preserving data flow known as the ‘separation principle’ [20]. Under this principle, PII is split from clinical information, with only PII (no clinical data) provided to linkage units and clinical information (no PII) provided to the end-user [9, 21].

Despite these technological and information privacy strategies, a broader consumer consultation associated with data utilisation is largely overlooked [22, 23]. There is however growing evidence for consumer support when sharing administrative health data [22–26], with an increased willingness to participate in health research involving the sharing of de-identified data rather than personal information [27]. In a recent survey, 90% of Australians were willing to share their de-identified health data for medical research and to improve healthcare [28]. Furthermore, studies have shown consumers in Australia [29], UK [29–32] and the United States [30] have a genuine desire to share their data for research if it can be used to help improve health outcomes, advance health service delivery and drive health policy [33]. However, this willingness is often limited by concerns from both patients [29, 31, 32, 34] and clinicians [35], which predominantly relate to privacy, trust and transparency; with a strong preference to maintain individual control of who has access to the data and the purpose of its use.

In Australia, much of the existing consumer engagement on this issue has been gathered through qualitative approaches to validate a range of overlapping factors influencing the willingness for data-sharing [22, 27]. However, it is uncertain how these factors are related or impact each other. Stated preference methodologies have increasingly been used internationally to evaluate various aspects of decision making in healthcare [36–38] including service utilisation [37], quality improvement [39] and data sharing [25, 26, 40]; providing respondents with a choice between two or more possibilities allowing for the quantitative estimate of preference between choices. Our study utilises this approach to provide a clearer understanding of the factors influencing the willingness of consumers to share their health data for clinical, health policy and research purposes and to ascertain the relative importance associated with each attribute. Consequently, this information will identify challenges and opportunities around establishing data collection systems and help develop communication with consumers to improve their confidence in the processes and willingness to participate in data linkage programmes.

## Methods

This study used an online stated preference questionnaire, based on discrete choice experiment (DCE) best practices [41], to quantitatively evaluate a set of factors influencing public preferences. In each choice set, the choice was between being willing to share data or not (i.e., an opt-out) and designed to reflect real-world decision-making [42]. Paired comparisons with or without an opt-out were considered but were not used as they did not reflect realistic decisions that might be made by community members.

The survey was circulated to a panel of Australians over the age of 18 who received reimbursement on completion of the survey. Prior to initiating the online survey, each respondent consented to participating in the online survey which consisted of four parts: socio-demographic questions; an introductory narrative for eligible respondents; choice-set questions contributing to the DCE; and a standard survey relating to each of the factors, which was not utilised in this study.

Socio-demographic questions included categorising participant’s age, gender, indigenous status, remoteness, an index providing a measure of a region’s socio-economic advantage and disadvantage (SEIFA) [43], income and education levels, including confidence and familiarity with personal health management and Australia’s personally controlled integrated electronic health summary known as My Health Record [44]. Example choice set questions are available in the (data in S3 Appendix), with choice-set questions and attributes described below.

### Ethics approval and consent to participate

The survey was carried out in accordance with relevant guidelines and regulations. The research received ethics approval from the Curtin University’s Human Research Ethics Committee (Ref approval number: HRE2019-0619-011), with all participants providing informed consent to participate.

### Conceptualising the stated preference

Experimental design, distribution and analysis were informed by previous studies [41, 45–48]. The approach utilised an integrated formative research design to ascertain potential attributes and levels. An initial literature scan of previous research investigating consumer perceptions and attitudes to sharing medical data for secondary use [25, 29, 31, 32] was conducted and summarised by the research team. The summary of data-sharing attributes was reviewed by an Advisory Group made up of consumer group advocates and researchers experienced in DCE methodologies, including those involved in data linkage, to assess attribute appropriateness and those that influenced preference. This was followed by two online public participatory group discussions in the form of focus groups [26, 49, 50], conducted between July and September 2021 to iteratively validate the attribute choices and to ensure appropriate and accessible language was used.

A total of 15 individuals attended one of the two sessions (n = 7, n = 8). Participants were recruited by the author primarily through consumer advocacy support group discussion forums as part of a broader study [51]. Interested participants registered for the event in an online registration portal and were subsequently contacted by a member of the study team to inform them of the purpose of the study, reviewed the consent form, and booked a convenient focus group time. At the time of contact, the study team employed the snowball subject recruitment technique [52], asking participants to recommend others they thought would be interested in participating in the study.

From these focus group sessions, the study team iteratively validated the attributes influencing preference with an emphasis on providing reasonable and appropriate trade-offs so as not to introduce hypothetical bias [53, 54]. Participants were encouraged to improve the wording of the choice set questions for clarity and understanding and were provided opportunities to suggest additional factors they felt would be important to include in the stated preference survey instrument. No additional factors were identified.

### Survey instrument

As a result, five unique attributes, with an average of five different levels were identified. The final discrete choice set included the following attributes: 1) individuals or institutions who will have access to data for analytical purposes (i.e., analysing group); 2) type of information used for data integration; 3) purpose for integration; 4) information governance, and 5) anticipated benefit (See Table 1)

**Table 1:** Attributes and levels.

Attribute	Levels
**A) Analysing Group:** **The individuals or institutions who will have access to the data and be doing the analysis**	The analysing authority should only include: 1. University researchers ^+^ 2. Healthcare providers involved in your care 3. National and State Department of Health researchers 4. Private companies (e.g., insurance analysts, market researchers, grocery retailers and technology companies [Google, Amazon, Microsoft])
**B) Type of information:** **The types of information you will be required to provide**	The types of information used should only include: 1. Information from your General Practice records linked with information from your other health records, including other General Practices and health service providers (e.g., radiology, physiotherapy, specialists, pathology and hospital records) 2. Information from your health records linked with your Medical Benefits Schedule Medicare* services subsidised by the Australian Government and Pharmaceutical Benefits Scheme** (prescription medication) information ^+^ 3. Information from your health records linked with third-party information (e.g., grocery store loyalty cards) 4. Information from your health records linked with insurance records
**C) Purpose:** **The reason for the data to be integrated or linked**	Research using linked health data should be: 1. Only if it benefits the individuals whose information is being collected 2. Only if it provides general public health benefits ^+^
**D) Information governance:** **The storage and use of provided information will be guided by the regulations and policies stipulated by the following organisations**	The information and processes should be managed by: 1. The organisations undertaking the research ^+^ 2. Relevant public health service (Western Australian Department of Health) 3. A non-governmental, independent body 4. The Australian Government
**E) Anticipated Benefit:** **By participating the proposed integrated health system, it is anticipated to have the following benefits**	Anticipated improvement from the data linkage program: 1. Improved and/or maintained health outcomes 2. Reduce patient wait times for practitioner and specialist consults ^+^ 3. Lower patient out of pocket costs for health services 4. An easier system to navigate (reduction in unnecessary tests, better access to your information- reduced duplication of repeating information

* Medical Benefits Schedule (MBS): Information related to general practitioner services, diagnostic tests, therapeutic procedures and specialist visits.

** Pharmaceutical Benefits Scheme (PBS): Records containing dispensed medications subsidised by the Government.

^+^ Used as a reference in the analysis

Based on these five attributes, we used the full-factorial of 512 possible combinations in our respondents enabling every possible combination to be seen, thereby eliminating any restrictions or blocking of choice set combinations. Each respondent was allocated 12 of the possible combinations, selected at random, a number similar to previous healthcare studies [48].

The number of choice tasks per person was derived through a careful consideration of balancing the statistical efficiency and the cognitive burden on the respondents. Additionally, the use of randomised task allocation among respondents reduces bias, as respondents are not all answering the same set of questions. This allocation strategy allows for the construction of an efficient experimental design, capturing a wide array of responses across different combinations of attributes and levels.

The discrete choice sets presented were randomised for each respondent, with all 512 possible unique combinations being presented at least 12 times throughout the course of the experiment. Prior to commencing the choice set questionnaire, respondents received detailed information on all attributes and levels, as well as an example choice set and instructions on how to complete it. Respondents were then given a choice set scenario and asked whether they would or would not be willing to share their data.

The survey was paused and tested after receiving the first 50 responses to validate the data collection process. Preliminary analysis of these responses indicated that no modifications to the survey instrument were required as regression coefficients and other responses were largely as expected.

### Sampling frame

The survey ran from 18^th^ of October to the 23^rd^ of November 2021. Using a third-party private survey sampling company, the survey was distributed to a representative sample frame of Australians, indicative of age (≥ 18 years), gender and location with no exclusions. Consented participants answered a series of screening questions as part of the eligibility assessment process.

The use of randomised task allocation among respondents was used to reduce respondent bias preventing respondents are not all answering the same set of questions, allowing for the construction of an efficient experimental design, capturing a wide array of responses across different combinations of attributes and levels. Additionally, prior to commencing the questionnaire, respondents were provided information on all attributes and levels, as well as an example choice set and instructions, further mitigating potential sources of bias. The application of a diverse set of sampling approaches was implemented to obtain a representative set of generalisable responses.

### Statistical analysis

Analysis was conducted using python version 3.5 [55] which included the following libraries (pandas, numpy, scikit-learn and matplotlib), R version 4.0.3 (2020-10-10) [56] and Stata/SE V14.0. Logistic regression was used to quantify the impact of the independent variables on respondent choice. Levels deemed to be least objectionable were selected as the reference variable for each of the attributes. Each attribute was dummy-coded transforming our categorical variables into a binary numeric format relative to the base level enabling multiple groups to be represented by a single regression equation and used for interpreting the regression coefficients.

Additionally, dummy coding facilitated interpretation of the model coefficients reflecting the mean difference in the latent dependent variable for a given category compared to the reference category (coded as ’0’), enabling interpretation of how different factors influence the public’s preferences for sharing health data.

The results of the logistic regression (Table 3) summarise the overall analysis enabling the assessment of each attribute and its associated levels. Determining the importance of each attribute and the different levels the attributes can take relative to other attributes, was made by estimating the difference each level and corresponding attribute made to the total utility. The constant reflects average preferences for the omitted levels in each of the attributes, and every coefficient is relative to the omitted level in that attribute. Therefore, the inferences around (for example) statistical significance are sensitive to the choice of the omitted levels and were considered in that context. An assessment of response heterogeneity to determine the effect of response similarities associated with the presented attributes and levels was conducted to identify unobserved (latent) clusters of respondents’ preferences.

### Subgroup analysis

To ascertain the willingness of consumers to participate in data-sharing programmes, the study cohort was stratified by demographic categories, ordered by the proportion of respondents who answered yes in descending order (data in S2 Appendix) and summarised in Table 4. An intra-population subgroup comparison demonstrated the order of willingness between the subgroups to participate in data-sharing activities. The percentage from each of the contributing population subgroups was compared to obtain the order of likelihood within the study cohort and their willingness to share data under the omitted levels. Proportions were used as they are not sensitive to the choice of omitted level in the regression.

## Results

A total of 1,138 individuals completed the Qualtrics survey and were required to complete 12 choice sets. After exclusion of ‘speeders’ (described below) this yielded a total of 12,036 observations. A targeted sampling approach was used to reflect the Australian population by both age and gender as identified by the Australian Bureau of Statistics [57]. Although sample sizes across healthcare-related stated preference studies have been shown to vary considerably [58], recent national studies with a similar focus utilised a final sample set of between 465 [26] and 1004 [25], suggesting a comparable cohort.

Respondents who completed the survey under the minimum estimated time of 2.5 minutes (n = 135 (11.9%)), also referred to as ‘speeders’ [59] suggested low levels of engagement with the survey [59] and were subsequently excluded from the analysis. The remaining respondents (n = 1,003) completed the survey with a median time of 7 minutes, which was comparable to similar studies [26].

A comparison to the Australian population (Table 2) showed that our study cohort may not necessarily be generalisable with lower proportions of older Australians (65 and older) participating in the survey (16.7% of respondents compared to 23.6% of the population) with lower male response rates for younger age group; higher male respondent rates for older participants, and 78% (compared with 72% of Australians) living in metro regions [60].

**Table 2:** Study sample characteristics, comparing sampling frame versus participants who completed the DCE.

	Sampling Frame		Completed Participant Quota (N = 1,003)		
Age Group (years)	Percentage of adult Australians	Gender ratio as June 2021 from the ABS (Male: Female)	Male	Female	Percentage
**18–24**	10.71	1.06	21	56	7.72
**25–34**	18.08	1.00	59	127	18.66
**35–44**	17.11	0.98	86	134	22.07
**45–54**	15.84	0.98	80	106	18.66
**55–64**	14.65	0.96	94	68	16.25
**65–74 and over**	23.61	0.78	139	27	16.65
**Sub total**	100.00	-	479	518	100.00

Total population of Australia from all ages was 25,688,079 62]

### Heterogeneity of respondents

**Fig 1:**
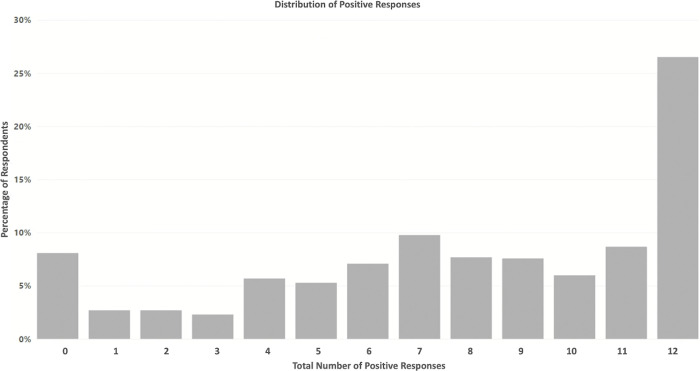
Distribution of the total number of positive responses to the choice-set questions.

The distribution of the total number of positive responses, representing the willingness to share data, is shown in Fig 1.

**Fig 2:**
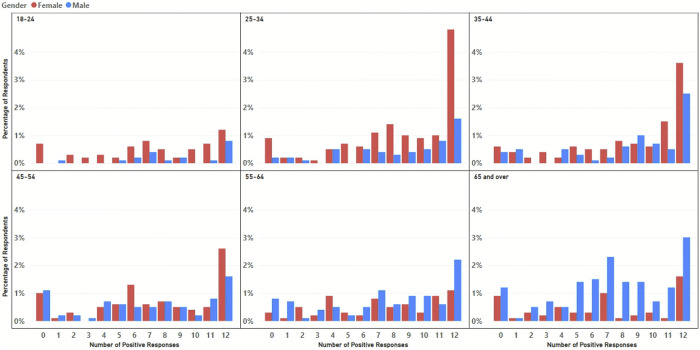
Distribution of the total number of positive responses to the choice-set questions for females (red) and males (blue).

Of the participants who completed the survey, 26.5% of respondents always answered Yes, while 8.1% always selected to not provide data. Further demographic analysis Fig 2 indicated females aged between 25–44 years, and males older than 65 years were more likely to answer yes to all choices, suggesting these groups are willing to share data under any conditions. Conversely males aged 45 years and over were more likely to say no, suggesting concerns about participating in data-sharing programmes.

### Choice set analysis

Results from the logistic regression analysis (Table 3) were statistically significant (p<0.01) across most attribute levels. Positive coefficient values reflect a preference for a choice (level) compared to the reference variable of each attribute.

**Table 3:** Summary of logistic regression (for the complete dataset refer to data in S1 Appendix).

Dimensions	Coefficient	Standard Error	p-value
**Analysing Group**			
University Researchers	0	Reference	
Healthcare providers	0.231	0.061**	0.000
National / State Health Departments	0.156	0.061**	0.010
Private Companies	-0.793	0.067**	0.000
**Health Record Linked With**			
General Practice / Primary Care	-0.075	0.058	0.196
Government subsidised programmes (MBS/PBS)	0	Reference	
Third Party	-0.597	0.061**	0.000
Insurance	-0.254	0.061**	0.000
**Purpose**			
Personal benefits	0.033	0.040	0.413
Public benefits	0	Reference	
**Governed & Managed By**			
Research Organisations	0	Reference	
State Health Department (Public health services)	0.084	0.058	0.143
Non-Government / Independent Organisations	-0.113	0.057 *	0.047
Nat. Health Department (Government)	0.104	0.056	0.062
**Anticipated Benefit**			
Improved health outcomes	0.074	0.055	0.184
Reduced Wait Times	0	Reference	
Cost reduction	0.137	0.057 *	0.016
Health system efficiencies	0.028	0.058	0.628
**Constant**	0.878	0.090 **	0.000

MBS = Medicare Benefits Schedule; PBS = Pharmaceutical Benefits Scheme

Statistical significance is indicated at the 5% level (*) and the 1% level (**)

The least preferred option was associated with having private companies analyse consumer health data. Instead, consumers preferred to have their health data retained within primary care and analysed amongst healthcare providers.

Of all presented attributes, respondents found information governance to be the weakest driver when considering whether to share their data. Consumers were more inclined for their data to be managed by the State health department (p<0.05) rather than non-governmental/independent organisations, with National/Federal government agencies representing a viable alternative.

Given the choice, consumers had no significant preference for the purpose of sharing data for either public or personal benefit. However, by sharing their healthcare data, consumers had an increased expectation of reduced costs (p<0.05)

### Subgroup analysis

On average, respondents across population subgroups were shown to be favourable to sharing their data for research purposes with over 49.6% of respondents agreeing to participate across all 11 categories, with the median (respondents who answered yes 50%>) shown in Table 4.

**Table 4:** Subgroup likelihood to participate in health data sharing-related activities, based on 50% of respondents who answered yes.

Category		Level [Number who answered yes > = 50%] Percentage		
Aboriginal or Torres Strait Islanders		No		Yes
		[700] 73%		[30] 86%
Health Record Familiarity		No	Yes	
		[433] 70%	[302] 79%	
Confidence in Managing Own Healthcare		Not Confident	Confident	
		[177] 67%	[558] 76%	
Age		> = 55		< = 54
		[263] 69%		[472] 76%
Education		Low [Y12/Certification]	High [Diploma/ Bachelor/ above]	
		[329] 71%	[390] 76%	
Chronic Condition		No Chronic Condition	With Chronic Condition	
		[382] 71%	[353] 75%	
Self-Assessed Health Rating		Poor	Excellent	
		[202] 71%	[533] 74%	
Region		Metro	Regional	
		[569] 73%	[166] 74%	
Gender		Male	Female	
		[350] 73%	[381] 73%	
SEIFA		Disadvantaged	Advantaged	
		[376] 73%	[359] 73%	
Income		Low	High	
		[<$84K]	[> = $84K]	
		[437] 74%	[265] 74%	

The cohort most willing to share their data were those that identified as Aboriginal and Torres Strait Islanders [61], which most likely is a reflection of the lack of cultural representation in the sample population and identified as a limitation below. Unlike their opposite cohort which showed a stronger preference for health data to be analysed within healthcare by providers and government health departments, those who identified as indigenous were considerably opposed (p<0.05) to the idea. Both groups however considered options in which their health records were both linked with insurance and third-party data and shared with private organisations for management and analysis to be the least attractive.

Respondents who identified as having a familiarity with the My Health Record and a high confidence in managing their own healthcare were found to be the second and third most willing subgroups to share their data respectively. As with the first subgroup, there was a comparable preferential aversion for data linkage with third-party data, however, a similar dislike of linking insurance data was not seen in the subgroup that was confident in managing their own healthcare. Individuals who were confident in managing their own healthcare had a significant reduction in preference for who could analyse their data, preferencing healthcare providers over government health departments (p<0.01). This subgroup also preferred to have their shared data governed and managed by either State or National health departments.

The fourth most willing subgroup to share data for research purposes were those who younger than 54 years of age. In contrast to other subgroups, younger respondents were found to have a significant interest (p<0.001) in having their health records linked with third-party datasets and for the analysis to be conducted by private companies. Moreover, this cohort had a preference (p<0.5) for improved health outcomes as an anticipated benefit of sharing data.

Respondents with a higher education were ranked fifth in terms of likelihood to share their data for research. This subgroup preferred to have their data governed and managed by national health departments as opposed to non-government organisations (p<0.05), while individuals with low education were generally opposed to all forms of data sharing for data linkage programs.

Subgroups pertaining to chronic condition and self-assessed health rating had a similar proportion contribution from a sub-group ranking perspective. Those with chronic conditions disapproved of having any organisation govern or manage their data other than a research organisation. Respondents that self-assessed their current health status as high (excellent, very good or good) were also more likely to share their data. Individuals with a perception of being healthy on one hand, disapproved of having their data linked with third-party and insurance data, and on the other preferred health providers, and State and National health departments to the analysis with the latter to govern and manage their shared data.

The subgroup of respondents who lived in a regional area were more likely to share their data for research purposes than those located in a metro region. This group had a similar preference profile as those who were confident in managing their healthcare but tended to prefer the National health department for data governance and management as opposed to the State equivalent. A comparison by metro and regional regions for attributes including analysing groups, data shared for data linkage or purpose showed no significant differences; a result supported by other studies [62, 63]. However, the metro region respondents were found to have a preference towards cost reduction (p<0.001) and for their information to be governed by national government agencies (p<0.05) as opposed to non-government / independent organisations (p<0.1).

Similar to the younger subgroup, females, were supportive of, yet diametrically opposed to their male peers, having their data linked with both insurance and third-party data (p<0.001). Females had a slight preference (p<0.1) for improved health outcomes while males preferred cost savings (p<0.05) from an anticipated benefit perspective.

The income and SEIFA subgroups had analogous profiles with the exception of the lower income subgroup displaying stronger support for data to be analysed by the National health department and an increased preference for cost reduction (p<0.05). In comparison, the SEIFA subgroup representing respondents in areas of socio-economic advantaged had a stronger interest in having their general practice / primary care data linked with their health data.

## Discussion

Despite the existence of similar investigations conducted in the Netherlands [64], Scotland [32], and Northern European countries [25, 26], this study is the first in Australia to apply a stated choice methodology to quantitatively examine consumer perceptions and ascertain the willingness to share health data and participate in data linkage projects for research purposes.

The most frequently chosen options for sharing data were predominantly risk averse in nature with respondents generally willing to share their healthcare data under the provision that their data is governed by governmental agencies and analysed by either healthcare providers or government health departments; with an overall preference for data to be linked with government and primary care collections rather than third-party and insurance data. Previous studies found a similar consumer preference for sharing data with public services [25, 26]. In contrast, however, the Australian cohort had an overall dislike of private companies analysing their health data or having their data linked with insurance data or third-party data such as grocery store loyalty information or health tracking data. Unwillingness to engage with private companies may in part be attributed to the lack of trust and transparency which would have otherwise been accounted for by government health departments and university researchers [29, 31, 65, 66].

This is in direct contrast to the low preference for use of data by private companies, suggesting a corporate disdain associated with generating a profit through patient data [31]. These observations may be perceived as private organisations being less transparent in how shared data will be managed and utilised. This lack of understanding of how consumer data is governed, collected, managed and used has been reported in previous studies [23, 26, 32, 34, 67].

Lack of public trust also appears to be an impediment to linking data, a preference aligned with previous studies conducted elsewhere [25, 29, 31, 32]. This marked aversion towards sharing with private companies and linking data with insurance data or third-party data was prominent in the Australian cohort compared with Scotland and Sweden [25] despite Australian’s lack of confidence in their public healthcare system in contrast to OECD countries [5]. Given the choice, respondents preferred to have their health data anonymously linked with general practice data and with national programmes such as the Medical Benefits Schedule and Pharmaceutical Benefits Scheme that subsidize medical services and prescription medications respectively to eligible Australian residents [68]. Considering these are directly associated with patient care, respondents may have felt a closer connection and prioritised these ahead of insurance and private companies.

Consumers are becoming increasingly aware of the value of their health data and how it provides insights into improving health systems, patient outcomes [23, 69]; and as a potential revenue source for commercial companies [27]. Consumers also have specific expectations on how and with whom their data is shared. Respondents strongly preferred to have their health data analysed by healthcare providers with preferences for government agencies and university researchers as second and third options respectively. Preferencing healthcare providers over other choices may be interpreted as trusting healthcare providers to better understand data and how it can be used (i.e., consumers feel as though they stand a better chance to benefit directly than if their data were to be analysed by another entity).

This finding was further reinforced from an information governance perspective with significant opposition (p = 0.047) towards Non-Government / Independent Organisations managing health data. This antagonistic observation may however be indicative of attribute misunderstanding, resulting in an immediate dismissal of the option without thorough evaluation of the presented alternatives. Prior research has demonstrated consumers misunderstanding of information governance [25, 29], which may complicate decision-making process in such contexts [61, 70, 71]. In designing the task choice, as much information as would be typical for a person making this kind of decision in the real world was provided. If the latter explanation is to be considered, it suggests the way information governance is explained in lay settings is inadequate to allow people to express a preference. Consequentially, further work should be done in terms of consumer understanding of data governance.

Furthermore, additional clarity on how data will be used and for what purpose may be required for consumers to feel comfortable with sharing their data. These attributes did not resonate with those that identified as having a low income, education and self-assessed health rating and were subsequently observed as being the least likely groups to share their data suggesting an individual health literacy component [72, 73] that may need to be considered when engaging for data sharing programs [62, 63].

While previous studies suggested an altruistic intent as a driver for sharing data for research purposes [27, 29, 32, 74], the choice set questionnaire showed consumers to be ambivalent about whether sharing data was for either public or personal purposes. Consumers were more likely to share their data if there was an anticipated benefit of reduced costs across the health system, a similar preference seen in other studies [29, 32].

More nuanced scenarios come to light across the various cohorts and subgroups providing insights into attribute preferences that need to be considered when engaging with these groups for data-sharing activities. Awareness of these subgroup preferences may identify opportunities to reduce health inequities by determining which cohorts are more likely to share their health data, this could also provide insights into potential digital divides [75, 76]. Research suggests that subgroups with higher education [62, 63] or income are more likely to have more developed IT advanced skills or access to technology and subsequently face fewer digital health literacy challenges [77] than those from culturally diverse minorities or from regional areas [78]. In comparison, our results indicate that younger individuals, with a higher education, diagnosed with a chronic condition, that are familiar with the My Health Record system and have a positive self-assessed confidence of managing their health are more likely to share their health data for research. This suggests that health literacy, self-efficacy in health management, and familiarity with digital health tools significantly affect health data sharing willingness, indicating that concerns about health and associated data governance implications may influence the desire to share data and participating in research studies.

Additionally, it was observed that the younger subgroup was very comfortable to have their data linked with third-party data assets and for the analysis to be conducted by private companies, and observation not seen in other studies. This willingness to engage with privately-owned organisations may partly be reflective of the younger cohort’s familiarity and comfort with the proliferation of technology and social change leading to the emergence and adoption of new standards and behaviours associated with data sharing [79, 80]. Although respondents will remain cautious due to the potential risks involved [81], considerations in how these risks are managed should be a transparent part of the data sharing program.

Although this is an Australian study, many of the conclusions could be generalised to other industrialised nations. The findings reported here are important to take into consideration when engaging with consumers to participate in research requiring data sharing and/or data linkage activities. Developing a transparent research data governance process would enable consumers to control how their data contributes to the proposed outcome, in line with the concept of contextual integrity [82, 83] in that information should be collected, used, and shared in a way that is consistent with the context in which it was obtained, and may improve participation and willingness to share data.

### Limitations

There are several limitations in our study that need to be considered. Firstly, focus group recruitment was considerably impacted by COVID-19 restrictions. Isolation requirements forced both sessions to be rescheduled and reconfigured from face-to-face events to online engagements resulting in a reduced number of participants. Secondly, it is important to acknowledge that focus group participants were selected for their lived experiences, as part of a larger study [51], and renumerated for their time. This may have resulted in a cohort of participants who were more inclined to support or had a special interest in research. The focus group sessions should not, therefore, be considered representative of the broader public’s views, but rather as a guide to the range of views used to assist with the development of the survey instrument. Thirdly, although the survey instrument was iteratively trialled and updated at each focus group for understanding and appropriate wording, it is important for the design of choice experiments to consider respondent understanding [61] and the range of cognitive and technical abilities among participants and to minimize the complexity of the choice task to ensure the validity and reliability of responses [84]. Incorrect measurement and weighting of outcomes may potentially have resulted in suboptimal findings and inadequate recommendations.

In terms of generalisability, efforts were made for the survey to have a response rate representative of the Australian population, using an online panel for recruitment may have inadvertently introduced a selection bias for respondents who were technologically capable suggesting that not all subgroups were adequately represented. While Aboriginal and Torres Strait Islanders had a similar representation in our dataset (3.5%) as is seen in the general population (3.2%) [85], the contribution from this important population cohort may have in part driven some of the variances in the subgroup comparisons. Further investigations into perceptions of this subgroup should be considered.

## Conclusion

The results indicate an underlying interest in participating in data-sharing and linkage activities as long as specific conditions are met. Although there is a growing body of evidence identifying the various factors that impact data-sharing preferences, little is known about how these factors influence public willingness to share their health data for research projects. This study is the first of its kind in Australia that identifies attribute preferences for sharing and the conditions that need to be considered to support research activities that require data sharing and data linkage activities. Our research highlights data governance, analysing of stakeholder groups, the type of data with which health records are linked and the anticipated benefits as four dimensions that require careful consideration when engaging with consumers to participate in data-sharing programmes. These findings contribute to the growing body of knowledge by providing an understanding of the factors involved and the impact they have on consumers when deciding to share their data for research-related activities. As this field continues to develop and the perceived risks of sharing health data evolve, further evaluation of key factors, including health and digital literacy and adequate governance will need to be undertaken and incorporated into the engagement with the public around research activities that require data sharing and data linkage.

## Supporting information

S1 Appendix Table 1 –Data analysis and evaluation. (PDF)

S2 Appendix Table 2 –Subcategories. (PDF)

S3 Appendix Choice set example. (PDF)
