# Supplementary material for: Public preference on sharing health data to inform research, health policy and clinical practice in Australia: A stated preference experiment
Source: PLoS One. 2023 Nov 16;18(11):e0290528. doi: 10.1371/journal.pone.0290528 (PMC10653479; doi:10.1371/journal.pone.0290528)
Supplement: S1 Appendix — (PDF) [file pone.0290528.s001.pdf]

Dimensions	Final (n=1,003)		
Analysing Group	Coeffient	Standard Erro	Significance
University Researchers	0	(omitted)	***
Healthcare providers	.2314189	.0610996	***
National / State Health Departments	.1561328	.0606712	***
Private Companies	-.7932647	.0672266	***
Health Record Linked With			
General Practice / Primary Care	-.0754821	.0583917	
Government subsidsed programs(MBS/PBS)	0	(omitted)	***
3rd Party	-.5965346	.0610323	***
Insurance	-.2539777	.0607687	***
Purpose			
Personal benefits	.0327442	.0400186	
Public benefits	0	(omitted)	
Goverened & Managed By			
Research Organisations	0	(omitted)	
State Health Dept. (Public health services)	.0842837	.0575448	
Non Government / Independent Organisations	-.112525	.0565763	**
Nat. Health Dept. (Government)	.1043864	.0558862	*
Anticipated Benefit			
Improved health outcomes	.0735752	.0554244	
Reduced Wait Times	0	(omitted)	
Cost reduction	.1366733	.0568081	**
Health system efficiencies	.0281671	.0581607	
Constant	.8776437	.0897047	***
Pseudo R2	0.0401		

Format of values displayed:
Coefficient (Coefficient) Standard Error
* = Low significance (p<0.1)
** = Medium significance (p<0.05)
*** = High significance (p<0.01)

Region						Gende
Metro (n=778)			Regional (n=225)			Males (n=479)		
Coeffient	Standard Err	Signi	Coeffient	Standard	Signif	Coeffient	Standard E	Significan
0	(omitted)		0	(omitted)		0	(omitted)	
.2219223	.0691602	***	.2590644	.131276	**	.3953377	.0900042	***
.1836256	.0682577	***	.0704811	.131131		.2888685	.0913854	***
-.7981847	.0746162	***	-.7833286	.153380	***	-.8837237	.100741	***
-.0843604	.0651662		-.0364364	.131028		-.1182557	.0889745	
0	(omitted)		0	(omitted)		0	(omitted)	
-.5674007	.0703158	***	-.6799764	.123211	***	-.609315	.0904736	***
-.2307834	.0674951	***	-.3247716	.137777	**	-.3041805	.0922102	***
.0325954	.044771		.0343446	.088388		.1006907	.0588997	*
0	(omitted)		0	(omitted)		0	(omitted)	
0	(omitted)		0	(omitted)		0	(omitted)	
.1014878	.0655859		.0282027	.119890		.1061732	.0839202	
-.106516	.0639892	*	-.1310268	.120545		-.2215502	.0825103	***
.127469	.0627077	**	.0197512	.123359		.1034178	.0776189	
.092674	.0623975		.0127437	.119914		.0043899	.0792744	
0	(omitted)		0	(omitted)		0	(omitted)	
.1836538	.0642632	***	-.0188615	.121135		.2123625	.0833693	**
.0434353	.0663186		-.0279371	.120426		-.0446043	.0841377	
.8540345	.1003527	***	.9552556	.19954	***	.8336444	.1311754	***
0.0407			0.0402			0.0571		

r			Age						
									Yes
			Young (n=623)			Old (n=380)			Identified as
Females (n=519)			<=54 years of age			>=55 years of age			co[illegible]
Coeffient	Standard	Signifi	Coeffient	Standard	Signif	Coeffient	Standard E	Signific	Coeffient
0	(omitted)		0	(omitted)		0	(omitted)		0
.0962864	.083812		0.1554619	.07444	**	0.36512	.1058737	***	.1951816
.0448552	.080288		0.108515	.07582		0.2423	.101651	**	.1697219
-.691719	.089228	***	0.5561491	.08221	***	-1.2034	.1185512	***	-.762452
.0439213	.077659		0.0055699	.07308		-0.2176	.0969973	**	-.0691022
0	(omitted)		0	(omitted)		0	(omitted)		0
.5813448	.083259	***	0.3806652	.07459	***	-0.981	.1047946	***	-.5162744
.2143407	.080794	***	0.0990057	.07410		-0.5262	.1057978	***	-.1696175
.0256716	.055409		0.0217725	.05147		0.03767	.0643882		.070857
0	(omittec		0	(omitted)		0	(omitted)		0
0	(omitted)		0	(omitted)		0	(omitted)		0
.0718933	.079848		0.1653328	.07273	**	-0.0304	.0953618		.1531992
.0271034	.078463		0.0464328	.07459		-0.2178	.0871175	**	-.0461504
.1190009	.079401		0.1295899	.07159	*	0.07298	.0889372		.1947811
.1342313	.078776	*	0.1474479	.07295	**	-0.0383	.0873085		.0660241
0	(omitted)		0	(omitted)		0	(omitted)		0
.0607652	.078382		0.1280911	.07200	*	0.15261	.0947717		.1499752
.0922265	.081060		0.0693644	.07386		-0.0394	.0960826		.0617764
.9174658	.124545	***	0.7783904	.11397	***	1.05221	.1459026	***	.7265201
0.0285		***	0.0205	0	***	0.0888		***	0.0363

Chronic Condition								Education
(n=468)			No (n=535)					
having a chronic ndition			Did not identify as having a chronic condition			Low (n=463) Year 12 / Certifications		Diploma, Adv
Standard E	Signifi	Coeffient	Standard E	Signifi	Coeffient	Standard	Signifi	Coeffient
(omitted)		0	(omitted)		0	(omitted)		0
.0805731	**	.270173	.0937416	***	.2708786	.0875703	***	.196712
.0818987		.1355493	.0902682	**	.0279126	.0863859		.297761
.0932575	***	-.8350516	.0973729	***	-.7351222	.0997467	***	-.8476345
.078247		-.0845092	.087992		-.1381383	.0876257		.0015251
(omitted)		0	(omitted)		0	(omitted)		0
.0803721	***	-.6905175	.0936558	***	-.6275754	.0906022	***	-.6097081
.0785407	**	-.3528512	.094369	***	-.2559414	.0905799	***	-.2601045
.05463		-.0101929	.0591321		.0017965	.0580347		.0371908
(omitted)		0	(omitted)		0	(omitted)		0
(omitted)		0	(omitted)		0	(omitted)		0
.0780016		-.0057272	.0848521	**	.0652516	.0854361		.090357
.0774389	**	-.1984257	.082608		-.0835222	.0846319		-.1556676
.0741788	***	-.0088621	.0836265	**	.034218	.0822422		.1624968
.0758005		.0798066	.0813894		.1361782	.0818892	*	.0545927
(omitted)		0	(omitted)		0	(omitted)		0
.0756815		.1201995	.0857716	**	.1343829	.083253		.1761987
.0778439		-.0151204	.0875832		.0588163	.0847147		.0454759
.122537	***	1.068679	.1306783	***	.8372872	.1392167	***	.9309682
0	***	0.046	0	***	0.0353			0.0487

		Income				
High (n=515)						
anced Diploma & Bachelor and above			Low (n=588) < $85,000			High (n=357) >=$85,000
Standard Erro	Significance	Coeffient	Standard Erro	Significance	Coeffient	Standard Erro
(omitted)		0	(omitted)		0	(omitted)
.0882287	**	.2542046	.0800886	***	.2392363	.1018164
.0866272	***	.1702579	.0845566	**	.2031795	.0947138
.09298	***	-.8286727	.0895794	***	-.8057369	.11133
.0824285		-.1243847	.0762896		-.0388484	.0982934
(omitted)		0	(omitted)		0	(omitted)
.0854427	***	-.7298525	.080368	***	-.4947756	.1040893
.0859617	***	-.3070392	.0833821	***	-.2448538	.0963466
.0572395		.0173103	.0509797		.0334062	.0693259
(omitted)		0	(omitted)		0	(omitted)
(omitted)		0	(omitted)		0	(omitted)
.0803777		.0408539	.076079		.0756988	.096684
.0783256	**	-.1262569	.0748164	*	-.1599719	.0936532
.0786348	**	.086415	.076244		.0844529	.0895666
.0769656		.1049796	.0723298		.0534353	.0922644
(omitted)		0	(omitted)		0	(omitted)
.0790196	**	.1734993	.0775135	**	.0960806	.091264
.0818401		.0555558	.076534		-.0401506	.0966444
.1205427	***	.9820059	.1185216	***	.9045306	.1455209
		0.0463	0	***	0.0407	0

	Self-Assessed Health Rating						C
							Lc
	Low (n=719) [Poor & Fair]			High (n=284) [Excellent, Very Good & Good]			[Not at a Confide C
Significance	Coeffient	Standard Err	Signif	Coeffient	Standard	Signific	Coeffient
	0	(omitted)		0	(omitted)		0
**	.2379125	.1156973	**	.2291003	.07195	***	.2212567
**	.0799485	.1166291		.1932371	.07088	***	.1958757
***	-.889527	.1293752	***	-.7581775	.07882	***	-.7793243
	-.1036768	.1106407		-.0675758	.06907		-.1040214
	0	(omitted)		0	(omitted)		0
***	-.6400654	.1136073	***	-.5750124	.07270	***	-.6963011
**	-.3371635	.1166595	***	-.2177455	.07144	***	-.3700875
	.1028281	.0726879		.0083836	.04809		.0554058
	0	(omitted)		0	(omitted)		0
	0	(omitted)		0	(omitted)		0
	-.0093084	.1109982		.1230394	.06768	*	.0147682
*	-.1197037	.1144454		-.1120554	.06503	*	-.1855603
	-.0775556	.1019909		.1797423	.06660	***	.06619
	.0498203	.0986904		.0842722	.06708		.0662368
	0	(omitted)		0	(omitted)		0
	.1596714	.100947		.1259753	.06849	*	.0915205
	-.0273027	.1092761		.050334	.06868		.0419271
***	.9084189	.171024	***	.8626083	.10569	***	1.066392
***	0.0462	0	***	0.0388	0	***	0.0426

:onfidence In Managing Own Healthcare							AT
›w (n=265)<br>ıll Confident, Not<br>ınt & Somewhat<br>:onfident]		High (n=738)<br>[Confident & Very Confident]			No (n=958)<br>Does not identify as Aboriginal and<br>Torres Strait Islander		
Standard Erro	Signifi	Coeffient	Standard Erro	Signifi	Coeffient	Standard Erro	Significance
(omitted)		0	(omitted)		0	(omitted)	
.0749498	***	.265614	.1056454	**	.2493835	.0627597	***
.0714264	***	.073130	.1155069		.1534743	.0623543	**
.0799866	***	-.837346	.1278235	***	-.8279197	.0689641	***
.0725779		.001460	.1003851		-.0816386	.0601626	
(omitted)		0	(omitted)		0	(omitted)	
.0742013	***	-.366467	.1074749	***	-.6092275	.0627659	***
.0726078	***	.015034	.1107748		-.2580593	.0619891	***
.0486267		-.022749	.0714915		.0314404	.0407266	
(omitted)		0	(omitted)		0	(omitted)	
(omitted)		0	(omitted)		0	(omitted)	
.0676988		.256833	.1103938	**	.0705776	.0589517	
.0657075	***	.071974	.1119325		-.1222933	.0577477	**
.0673465		.207067	.1015961	**	.0855602	.0570271	
.0636329		.091551	.112284		.0733274	.056292	
(omitted)		0	(omitted)		0	(omitted)	
.0673091		.252229	.1066039	**	.1272996	.0582271	**
.0688072		-.033385	.1094264		.0474921	.0598925	
.1083715	***	.407674	.1582405	***	.88341	.0921309	***
		0.0402			0.0428		

͞SI			Health Record Familiarity			
Yes (n=35) Identifies as Aboriginal and Torres Strait Islander			No (n=620) [Heard about it - know nothing / little Never heard of it]			[Know abo
Coeffient	Standard Err	Significance	Coeffient	Standard Err	Significance	Coeffient
0	(omitted)		0	(omitted)		0
-.6493802	.3244703	**	.2277543	.0756905	***	.2671971
.2065106	.3934991		.1706801	.0741778	**	.1531144
-.2333675	.4166699		-.7162349	.081889	***	-.9275696
-.3014149	.3068709		-.0456018	.0705993		-.1491402
0	(omitted)		0	(omitted)		0
-.7421995	.3552898	**	-.5433948	.0732358	***	-.7244422
-.6857182	.3526265	*	-.1923875	.0742418	***	-.3970496
-.1930019	.2961683		.0676609	.0495401		-.0240696
0	(omitted)		0	(omitted)		0
0	(omitted)		0	(omitted)		0
.6771971	.3448464	**	.116898	.0715692		.0215943
.0505862	.3682809		-.0656125	.0704081		-.1987019
.4341799	.3458989		.1747615	.0693368	**	-.0151974
.0919475	.331283		.0506862	.0689549		.1196746
0	(omitted)		0	(omitted)		0
.4060205	.3647279		.1135348	.0698366		.1787074
-.4413066	.2814235		.0028142	.0715628		.0579804
1.754511	.5135402	***	.6251215	.1134448	***	1.33495
0.0525			0.0349		***	0.0534

				SEIFA		
Yes (n=383)		Disadvantaged (n= 509)			Advantaged (n=4	
ut it a fair bit / very well]		SEIFA (1 & 2 & 3)			SEIFA (4 & 5)	
Standard Errc	Significance	Coeffient	Standard Errc	Significance	Coeffient	Standard Errc
(omitted)		0	(omitted)		0	(omitted)
.1070323	**	.2356234	.085753	***	.2254383	.0882659
.1080127		.1597365	.0865129	*	.1625181	.085448
.1187332	***	-.7855222	.0973487	***	-.8027634	.0933045
.1072668		-.167559	.0793449	**	.0193839	.0864612
(omitted)		0	(omitted)		0	(omitted)
.1104153	***	-.6466145	.0856165	***	-.5383803	.0877807
.1067884	***	-.2176356	.0848166	***	-.2921919	.0879065
.0695316		-.0002019	.0555806		.072192	.0577928
(omitted)		0	(omitted)		0	(omitted)
(omitted)		0	(omitted)		0	(omitted)
.0984209		-.0144561	.0795917		.1819755	.0838734
.0958038	**	-.1610143	.0795331	**	-.0560366	.0809343
.0954779		.0857462	.0819999		.1278687	.0764349
.0930064		.0676893	.0803376		.0643439	.0765383
(omitted)		0	(omitted)		0	(omitted)
.0984791	*	.0355878	.0791729		.2290973	.0811565
.1005036		.0325137	.0832957		.0205285	.0820361
.1460995	***	.957815	.1265192	***	.7932588	.1278104
	***	0.0399			0.0425	

89)
Significance
**
*
***
***
***
**
*
***
***
