# Supplementary material for: Public preference on sharing health data to inform research, health policy and clinical practice in Australia: A stated preference experiment
Source: PLoS One. 2023 Nov 16;18(11):e0290528. doi: 10.1371/journal.pone.0290528 (PMC10653479; doi:10.1371/journal.pone.0290528)
Supplement: S2 Appendix — (PDF) [file pone.0290528.s002.pdf]

Category	L<br>[Number who answere
Aboriginal or Torres Strait Islanders	No<br>[700] 73%
Health Record Familiarity	No<br>[433] 70%
Confidence in Managing Own Healthcare	Not Confident<br>[177] 67%
Age	>=55<br>[263] 69%
Education	Low<br>[Y12/Certification]<br>[329] 71%
Chronic Condition	No Condition<br>[382] 71%
Self-Assessed Health Rating	Poor<br>[202] 71%
Region	Metro<br>[569] 73%
Gender	Male<br>[350] 73%
SEIFA	Disadvantaged<br>[376] 73%
Income	Low<br>[<$84K]<br>[437] 74%

evel<br>d yes >=50%] Percentage	
	Yes
	[30] 86%
Yes	
[302] 79%	
	Confident
	[558] 76%
<=54	
[472] 76%	
High	
[Diploma/ Bachelor/ above]	
[390] 76%	
With Condition	
[353] 75%	
Excellent	
[533] 74%	
Regional	
[166] 74%	
Female	
[381] 73%	
Advantaged	
[359] 73%	
High<br>[>=$84K]	
[265] 74%	

Total Respondents That Answered Yes			Gender		
			Female		M
Requency of Answering Yes	Total Resp.	% Resp.	Total	%	Total
>=12 (100% of the time)	266	27%	149	29%	117
>=11 (More than 92% of the time)	353	35%	196	38%	157
>=10 (More that 83% of the time)	413	41%	226	44%	187
>=9 (More than 75% of the time)	489	49%	258	50%	231
>=8 (More than 67% of the time)	566	56%	298	57%	268
>=7 (More than 58% of the time)	664	66%	346	67%	317
>=6 (More than 50% of the time)	735	73%	381	73%	350
>=5 (More than 42% of the time)	788	79%	408	79%	376
>=4 (More than 33% of the time)	845	84%	437	84%	403
>=3 (More than 25% of the time)	868	87%	448	86%	415
>=2 (More than 17% of the time)	895	89%	466	90%	424
>=1 (More than 8% of the time)	922	92%	475	92%	442
Always Answered "No"	81	8%	44	8%	37
Total Respondents (Yes and No)	1003		519	52%	479
Absolute difference of those that ansered Yes more than 50% of the time			0.34151		

51.7%
47.8%
62.1%
37.9%
46.2%
51.3%
46.7%
53.3%
95.5%
3.5%
58.6%
35.6%
51.2%
48.8%
77.6%
22.4%
61.8%

	38.2%
	28.3%
	71.7%
	26.4%
	73.6%
Average	49.6%

	Age				Education				Chronic Conditio		
ale	<=54 Years of age		>=55 Years of age		Year 12 / Certifications		Diploma, Advanced Diploma & Bachelor and above		Identified as having a chronic condition		Did ident havi chr cond
%	Count	%	Count	%	Count	%	Count	%	Count	%	Count
24%	187	30%	79	21%	121	26%	140	27%	113	24%	153
33%	246	39%	107	28%	159	34%	187	36%	158	34%	195
39%	284	46%	129	34%	183	40%	220	43%	192	41%	221
48%	329	53%	160	42%	216	47%	262	51%	229	49%	260
56%	380	61%	186	49%	247	53%	306	59%	267	57%	299
66%	426	68%	238	63%	294	63%	356	69%	315	67%	349
73%	472	**76%**	263	69%	329	71%	390	**76%**	353	**75%**	382
78%	503	81%	285	75%	349	75%	422	82%	378	81%	410
84%	536	86%	309	81%	380	82%	446	87%	402	86%	443
87%	544	87%	324	85%	394	85%	454	88%	412	88%	456
89%	557	89%	338	89%	407	88%	468	91%	425	91%	470
92%	574	92%	348	92%	423	91%	479	93%	434	93%	488
8%	49	8%	32	8%	40	9%	36	7%	34	7%	47
48%	623	62%	380	38%	463	46%	515	51%	468	47%	535
	6.552				4.67				4.025		

n	ATSI				Income				SEIFA		
not ify as ng a onic lition	No		Yes		< $84,000		>=$84,000		SEIFA (1 & 2 & 3) Disadvantaged		SEIFA Advar
%	Count	%	Count	%	Count	%	Count	%	Count	%	Count
29%	250	26%	15	43%	164	28%	89	25%	145	28%	121
36%	332	35%	19	54%	217	37%	117	33%	193	38%	160
41%	388	41%	22	63%	249	42%	143	40%	217	42%	196
49%	459	48%	25	71%	291	49%	174	49%	246	48%	243
56%	534	56%	27	77%	336	57%	204	57%	287	56%	279
65%	631	66%	28	80%	396	67%	238	67%	336	65%	328
71%	700	73%	30	**86%**	437	74%	265	74%	376	73%	359
77%	753	79%	30	86%	462	79%	291	82%	396	77%	392
83%	806	84%	32	91%	497	85%	308	86%	426	83%	419
85%	829	87%	32	91%	513	87%	315	88%	440	86%	428
88%	854	89%	34	97%	530	90%	323	90%	452	88%	443
91%	879	92%	35	100%	547	93%	328	92%	463	90%	459
9%	79	8%	0	0%	41	7%	29	8%	51	10%	30
53%	958	96%	35	3%	588	59%	357	36%	514	51%	489
	12.65				0.09				0.263		

	Region				Health Record Familiarity				Self-Health Ratir		
(4 & 5) ıtaged	Metro		Regional		Heard about it - know nothing / little Never heard of it		Know about it a fair bit / very well		Poor & Fair		Exceller Good &
%	Count	%	Count	%	Count	%	Count	%	Count	%	Count
25%	210	27%	56	25%	134	22%	132	34%	70	25%	196
33%	274	35%	79	35%	196	32%	157	41%	99	35%	254
40%	325	42%	88	39%	232	37%	181	47%	112	39%	301
50%	385	49%	104	46%	280	45%	209	55%	127	45%	362
57%	443	57%	123	55%	328	53%	238	62%	145	51%	21
67%	518	67%	146	65%	389	63%	275	72%	177	62%	487
73%	569	73%	166	**74%**	433	70%	302	**79%**	202	71%	533
80%	615	79%	173	77%	464	75%	324	85%	217	76%	571
86%	659	85%	186	83%	502	81%	343	90%	233	82%	612
88%	678	87%	190	84%	517	83%	351	92%	41	14%	627
91%	698	90%	197	88%	539	87%	356	93%	254	89%	641
94%	719	92%	203	90%	557	90%	365	95%	260	92%	662
6%	59	8%	22	10%	63	10%	18	5%	24	8%	57
49%	778	78%	225	22%	620	62%	383	38%	284	28%	719
	0.642				9.012				3.004		

ıg	Confidence of managing own healthcare			
ıt, Very ı Good	Not at all Confident, Not Confident & Somewhat Confident		Confident & Very Confident	
%	Count	%	Count	%
27%	59	22%	207	28%
35%	84	32%	269	36%
42%	95	36%	318	43%
50%	115	43%	374	51%
3%	129	49%	437	59%
68%	154	58%	510	69%
**74%**	177	67%	558	**76%**
79%	192	72%	596	81%
85%	209	79%	636	86%
87%	214	81%	654	89%
89%	223	84%	672	91%
92%	234	88%	688	93%
8%	31	12%	50	7%
72%	265	26%	738	74%
	8.817			
