# Supplementary material for: Public preference on sharing health data to inform research, health policy and clinical practice in Australia: A stated preference experiment
Source: PLoS One. 2023 Nov 16;18(11):e0290528. doi: 10.1371/journal.pone.0290528 (PMC10653479; doi:10.1371/journal.pone.0290528)
Supplement: S3 Appendix — (PDF) [file pone.0290528.s003.pdf]

Appendix:

Participants were provided with a background from which to base their decision, enabling their choices to accurately reflect their preferences in relation to an integrated healthcare system.

The integrated healthcare system, as explained in the narrative, was described as a system that coordinates and consolidates various aspects of healthcare (including primary care, specialist services, and hospital services) to ensure that patients receive comprehensive and coordinated care. The potential benefits and drawbacks of participating in such a system were mentioned with benefits relating to improved communication between healthcare providers, more personalised and efficient care, and potential cost savings. Potential drawbacks included concerns about data privacy and a perceived loss of control over one's healthcare.

Participants were then asked to state their willingness to participate in this integrated healthcare system. The choice between:

"I would be willing to participate in the integrated health care system"

and

"I would not be willing to participate in the integrated health care system"

was intended to provide a clear binary decision, reflecting the participant's overall preference given the potential benefits and drawbacks described in the narrative.

Example choice sets are shown below:

Example Choice Set One:

**A) Analysing authority**<br><br>The individuals or institutions who will have access to the data and be doing the analysis	Only University researchers would have access
**B) Type of information**<br><br>It will be required of you to provide information	Information from your General Practice records linked with information from your other health records, including other General Practices, health service providers (e.g. radiology, pathology) and hospital records)
**C) Purpose**<br><br>The reason for the data to be integrated or linked	Research using linked data should only be allowed if it benefits the individuals whose information is being collected

**D) Information governance**<br><br>The storage and use of provided information will be guided by the regulations and policies stipulated by	The information and processes should be managed by the organisations undertaking the research
**E) Anticipated improvement**<br><br>By participating in the data linkage program, it is anticipated to have the following benefits	Improved / maintained health outcomes

**I would be willing to participate** in the integrated health care system	☐
**I would not be willing to participate** in the integrated health care system	☐

Example Choice Set Two:

**A) Analysing authority**<br><br>The individuals or institutions who will have access to the data and be doing the analysis	University researchers, health care providers, government researchers, insurance analysts or market researchers
**B) Type of information**<br><br>It will be required of you to provide information	Information from your health records linked with private sector information (e.g. grocery store loyalty cards)
**C) Purpose**<br><br>The reason for the data to be integrated or linked	Research using linked data should be conducted for any purpose
**D) Information governance**<br><br>The storage and use of provided information will be guided by the	The information and processes should be managed by the Australian Government

regulations and policies stipulated by	
**E) Anticipated improvement**<br><br>By participating in the data linkage program, it is anticipated to have the following benefits	Efficiencies with which to navigate the health system (streamlined quality of care)

**I would be willing to participate** in the integrated health care system	☐
**I would not be willing to participate** in the integrated health care system	☐
